# Supplementary figures and images for: KIC (ketoisocaproic acid) and leucine have divergent effects on tissue insulin signaling but not on whole-body insulin sensitivity in rats
Source: PLoS One. 2024 Aug 20;19(8):e0309324. doi: 10.1371/journal.pone.0309324 (PMC11335129; doi:10.1371/journal.pone.0309324)

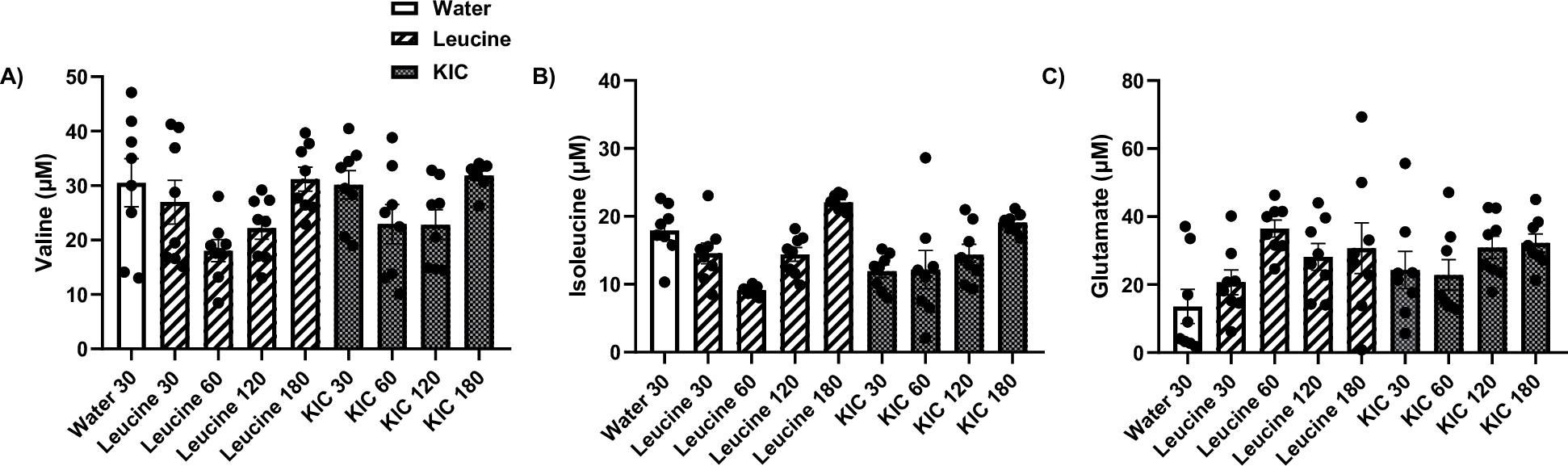

Supplement: S1 Fig — Rats were gavaged 0.75 mL/100 g body weight, twice with water, leucine (0.170 mM), or KIC (0.197 mM) 10 minutes apart. They were euthanized at different timepoints (30–180 min). HPLC was performed to measure valine (A), isoleucine (B), and glutamate (C) concentrations in plasma. Data are means ± SEM. N = 8 each group. (TIF) [file pone.0309324.s001.tif]

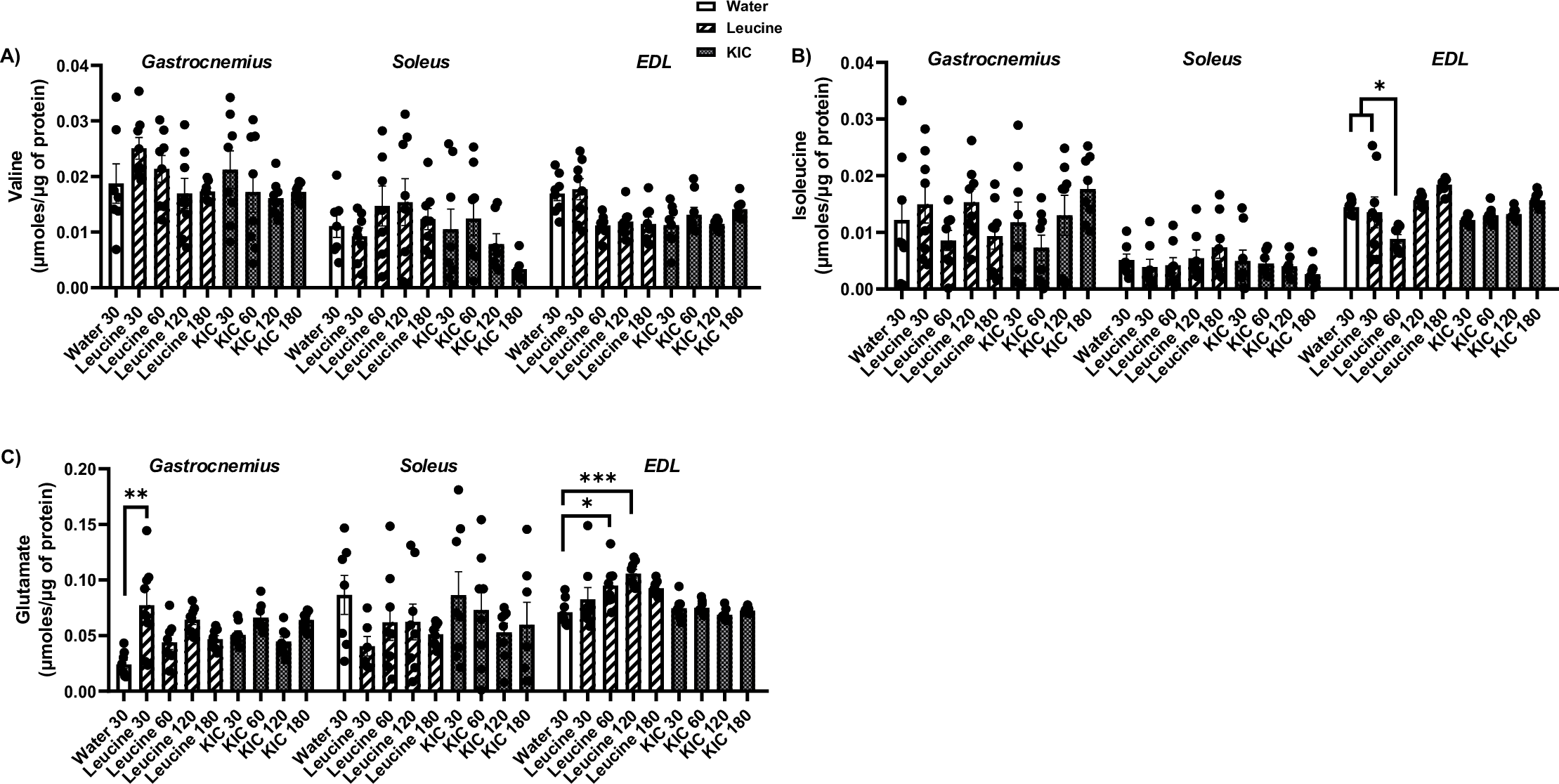

Supplement: S2 Fig — Rats were treated as explained in S1 Fig. HPLC was then performed to measure intracellular valine (A), isoleucine (B), and glutamate (C) concentrations in the gastrocnemius, soleus and EDL muscles. Data are means ± SEM. N = 7–8 each group; * p<0.05, ** p < 0.01, *** p < 0.001. (TIF) [file pone.0309324.s002.tif]

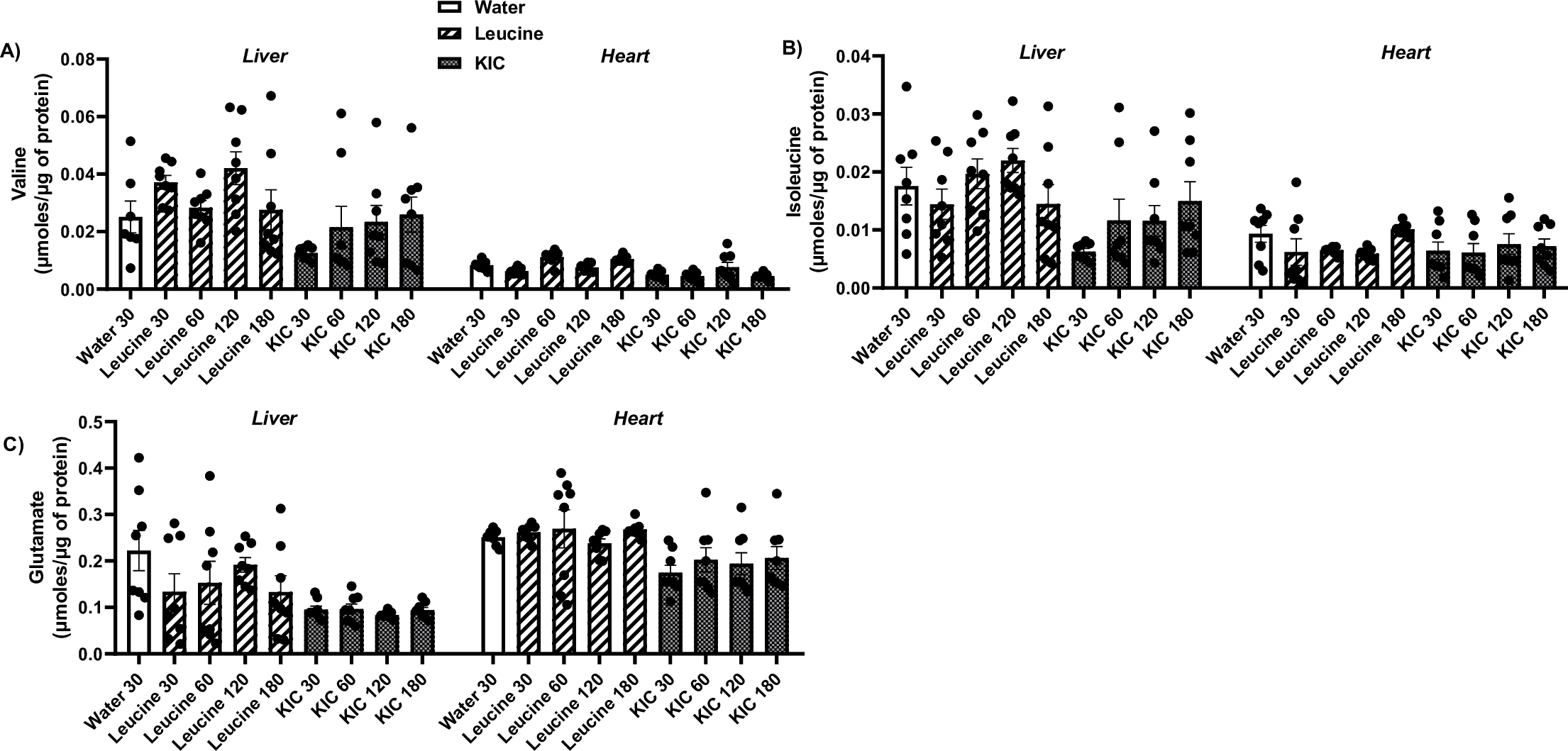

Supplement: S3 Fig — Rats were treated as explained in S1 Fig. HPLC was then performed to measure intracellular valine (A), isoleucine (B), and glutamate (C) concentrations in the liver and heart. Data are means ± SEM. N = 7–8 each group. (TIF) [file pone.0309324.s003.tif]
