# Supplementary material for: KIC (ketoisocaproic acid) and leucine have divergent effects on tissue insulin signaling but not on whole-body insulin sensitivity in rats
Source: PLoS One. 2024 Aug 20;19(8):e0309324. doi: 10.1371/journal.pone.0309324 (PMC11335129; doi:10.1371/journal.pone.0309324)

KIC (ketoisocaproic acid) and leucine have divergent effects on tissue insulin signaling but not on whole-body insulin sensitivity in rats

Gagandeep Mann, Stephen Mora, and Olasunkanmi Adegoke

Supporting Information

Uncropped and unadjusted western blots

Blots for fig 2c

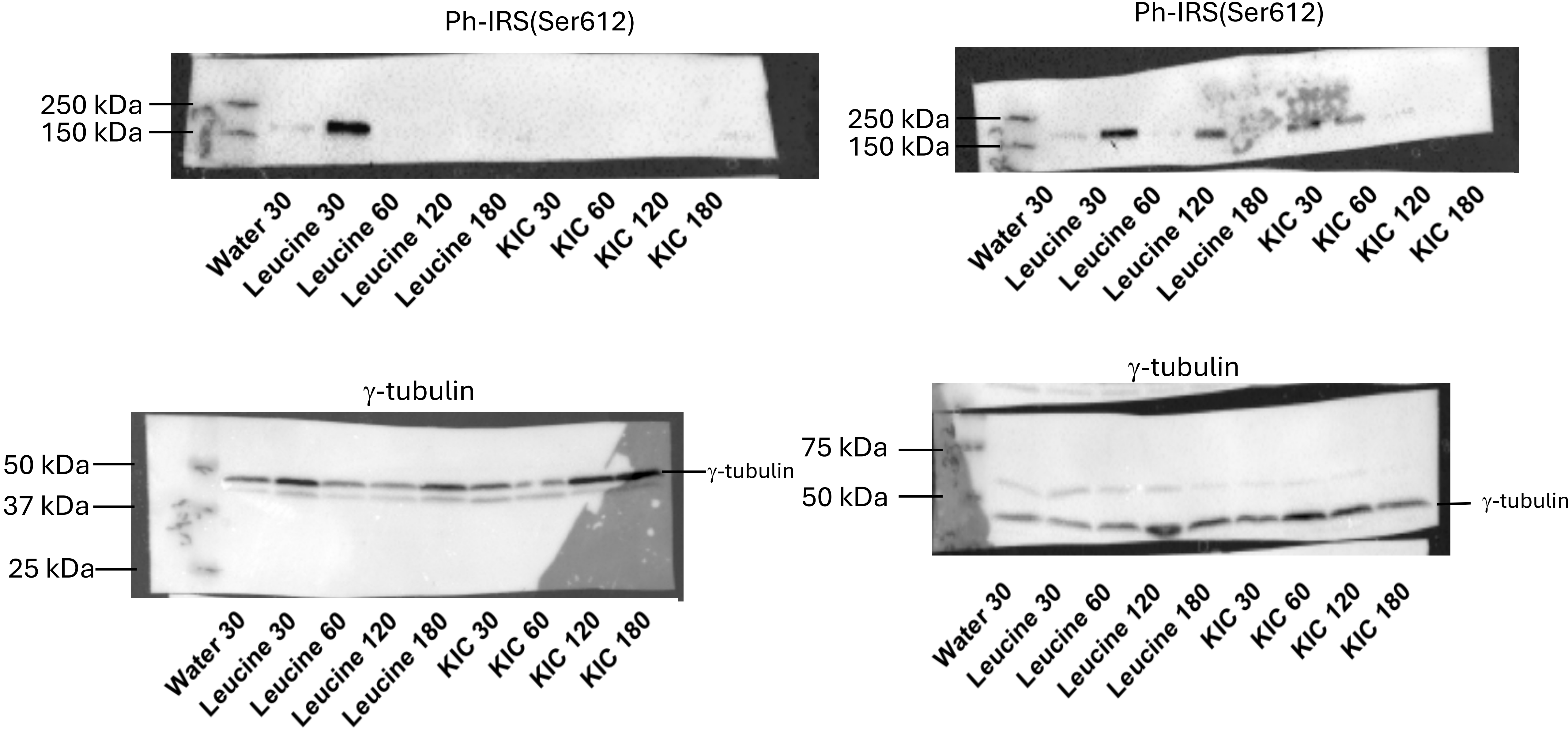

Blots for fig 2c

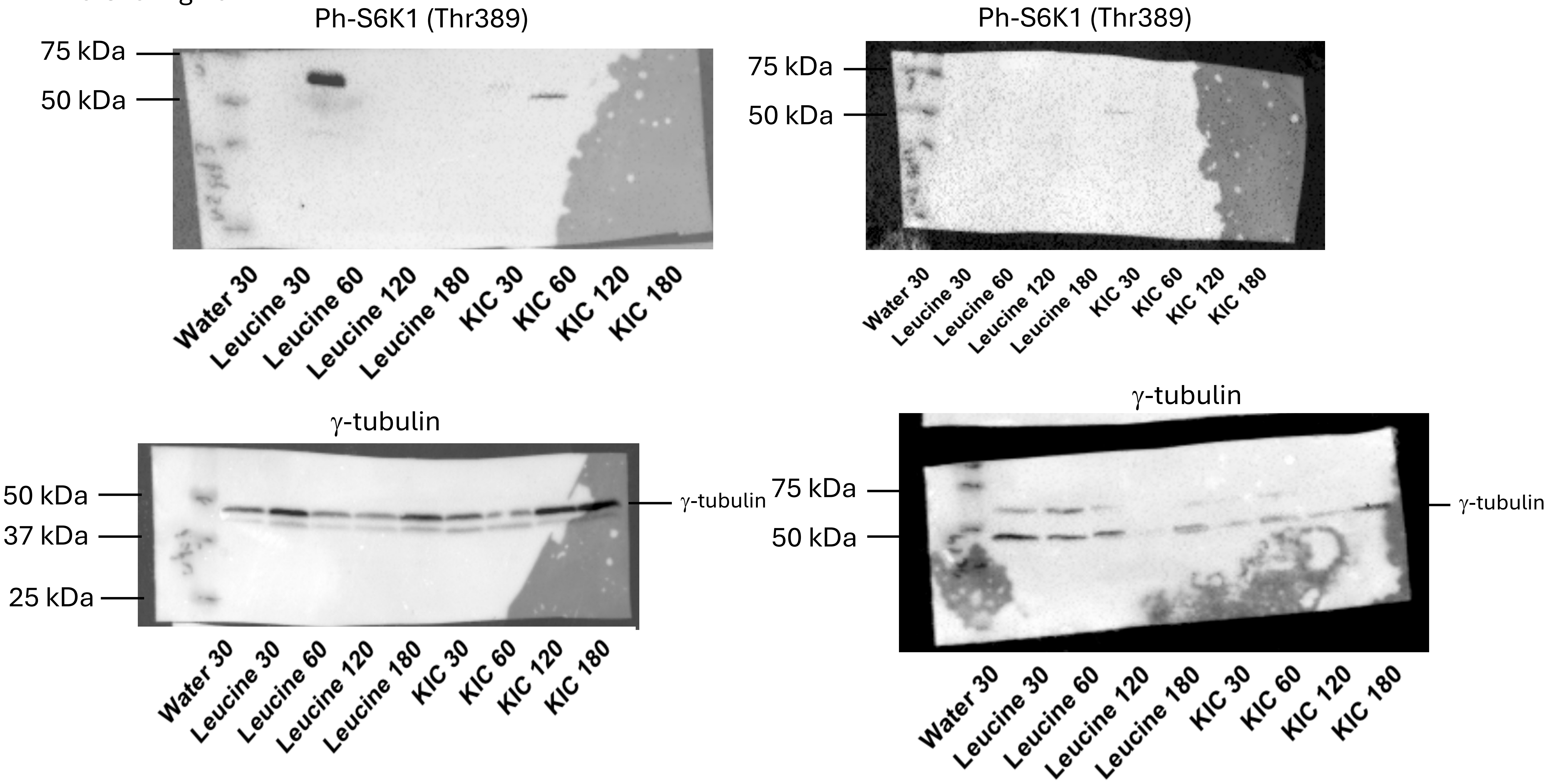

Blots for fig 2c

Ph-S6 (Ser235/6)

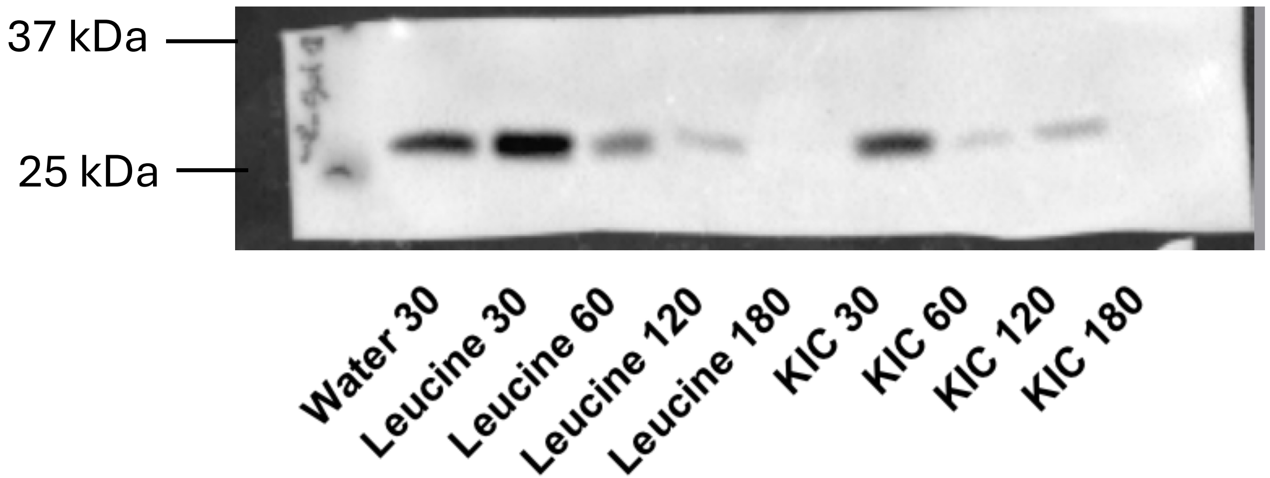

γ-tubulin

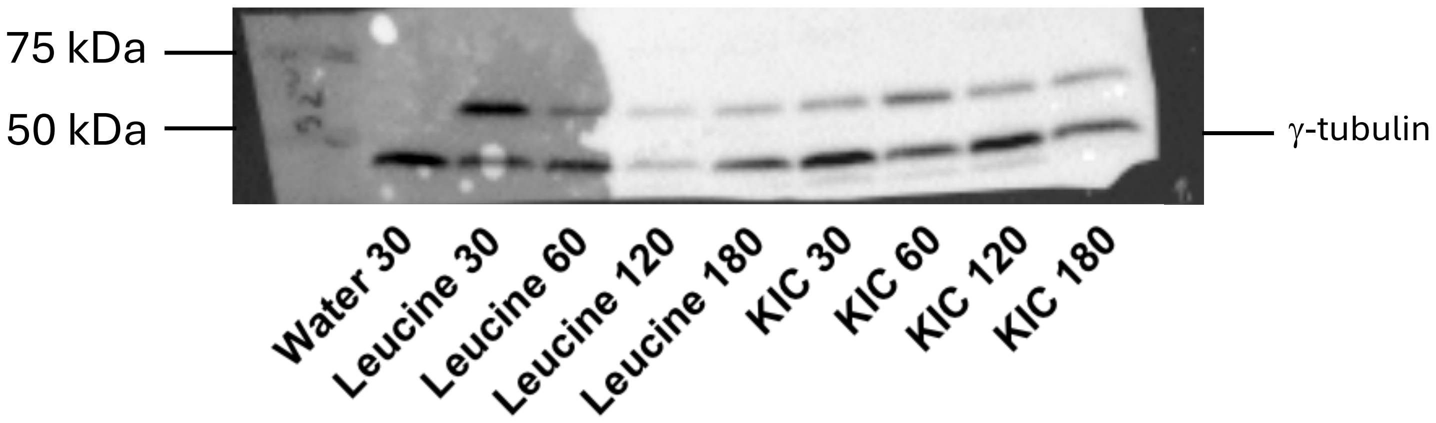

Blots for fig 2c

Ph-Akt (Ser473)

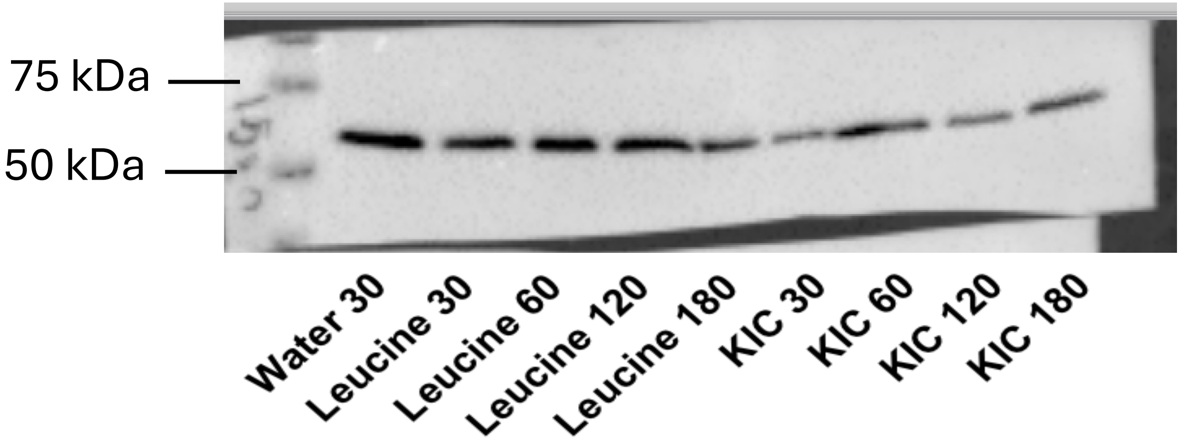

γ-tubulin

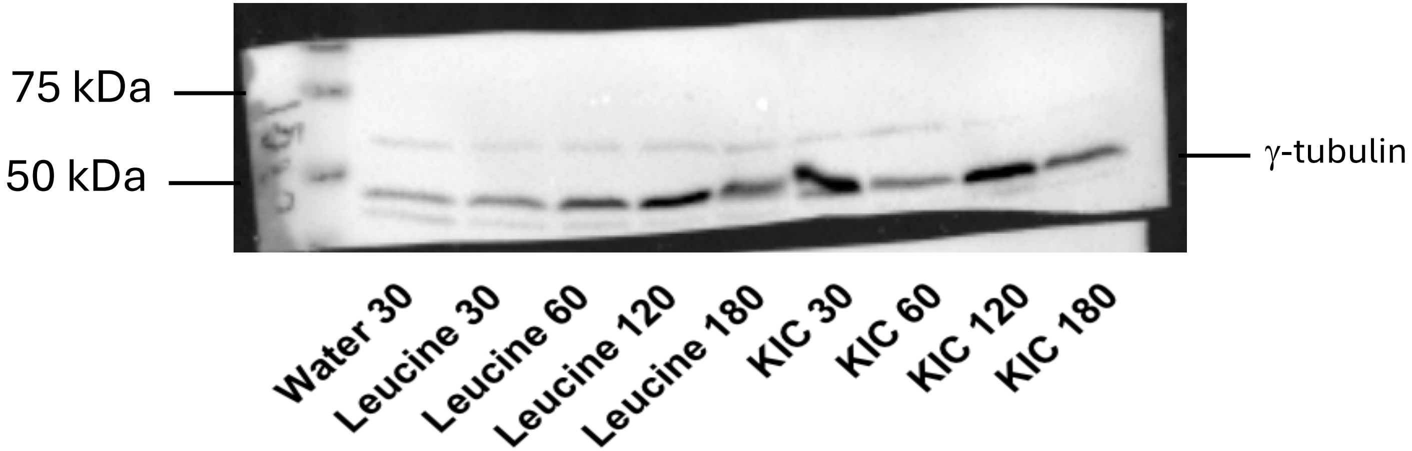

Blots for fig 2c

Ph-BCKD (Ser293)

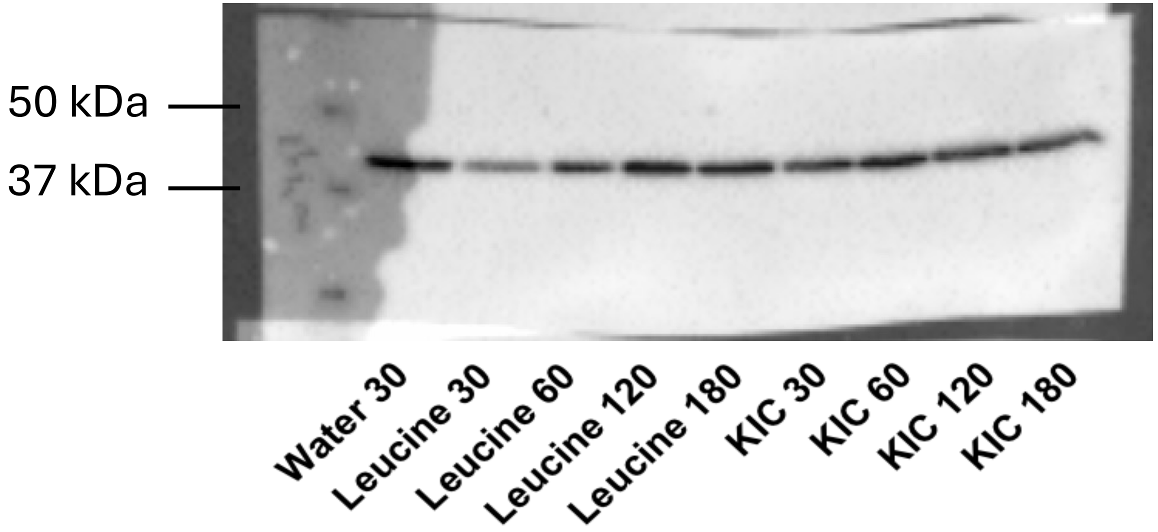

γ-tubulin

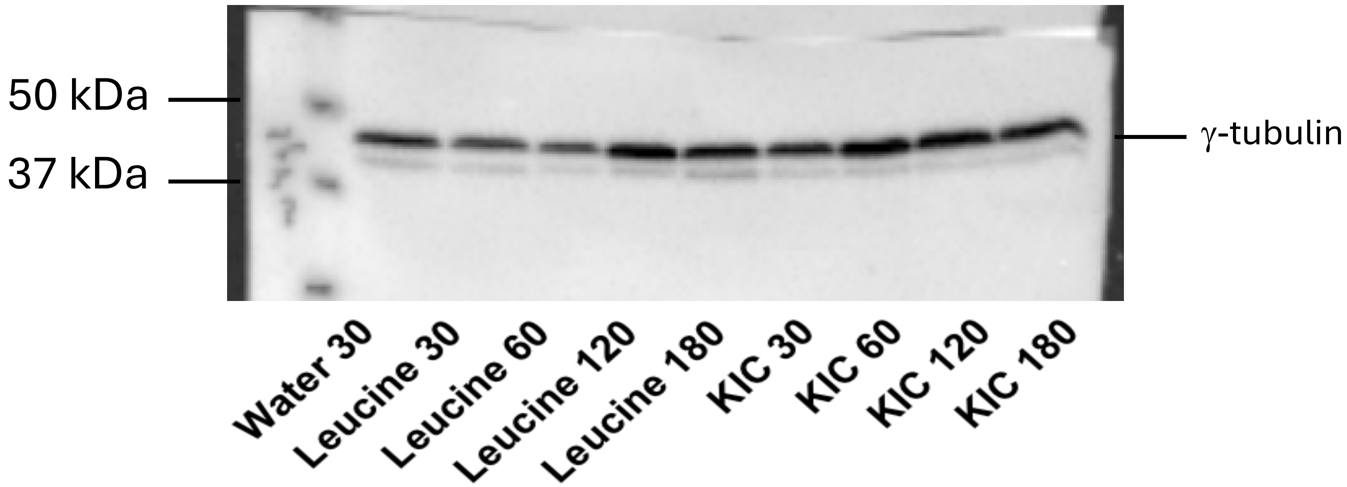

Blots for fig 3c

Ph-IRS(Ser612)

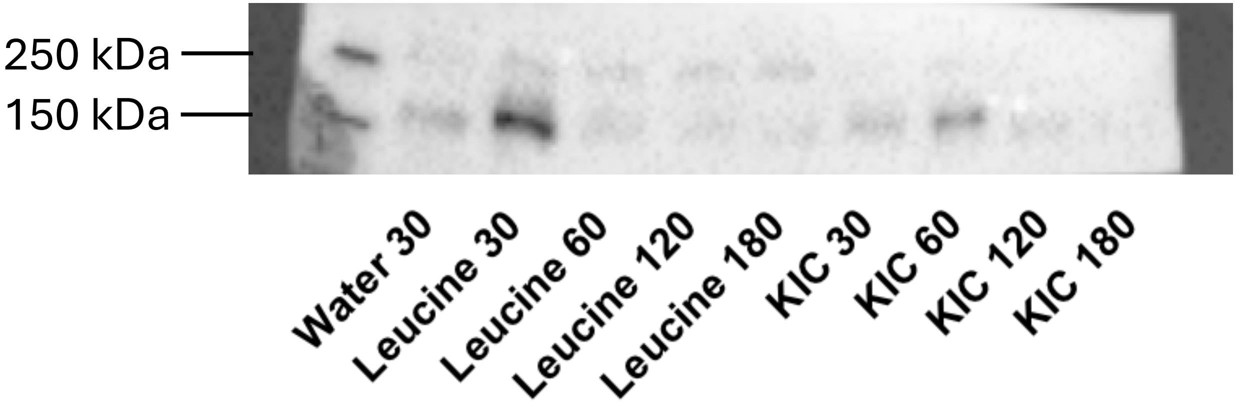

γ-tubulin

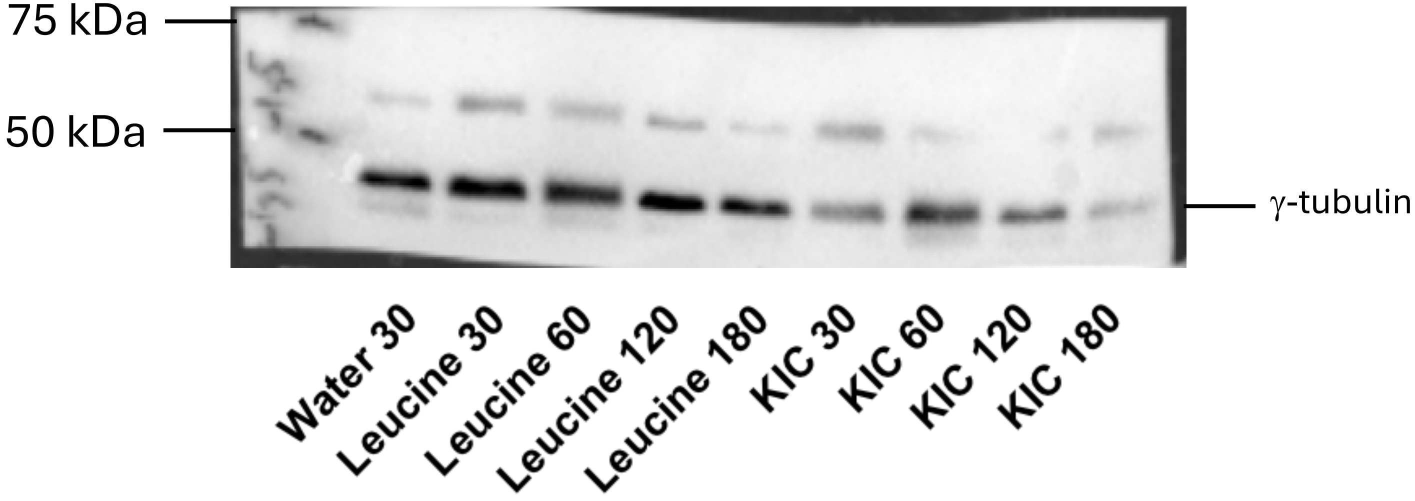

Blots for fig 3c

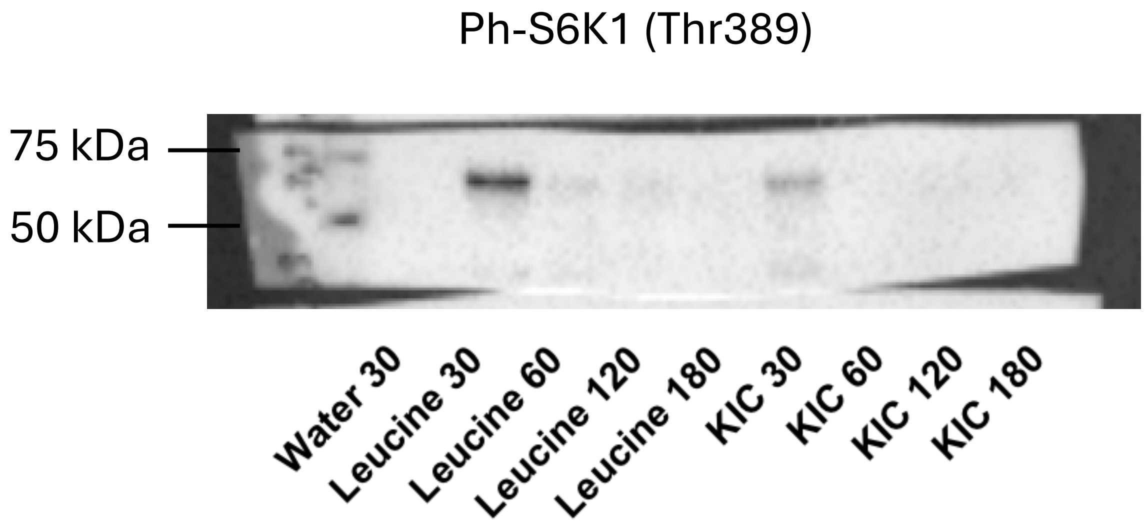

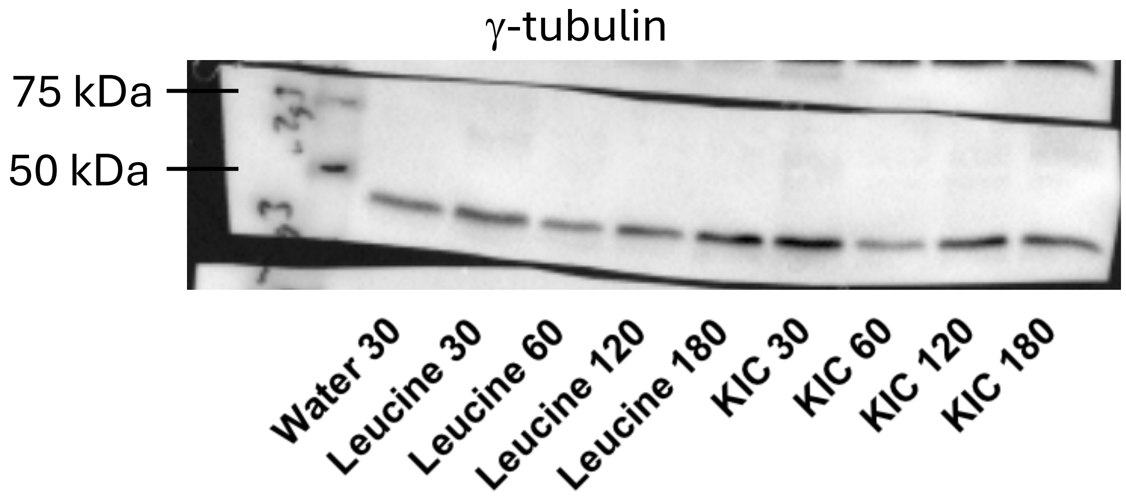

Blots for fig 3c

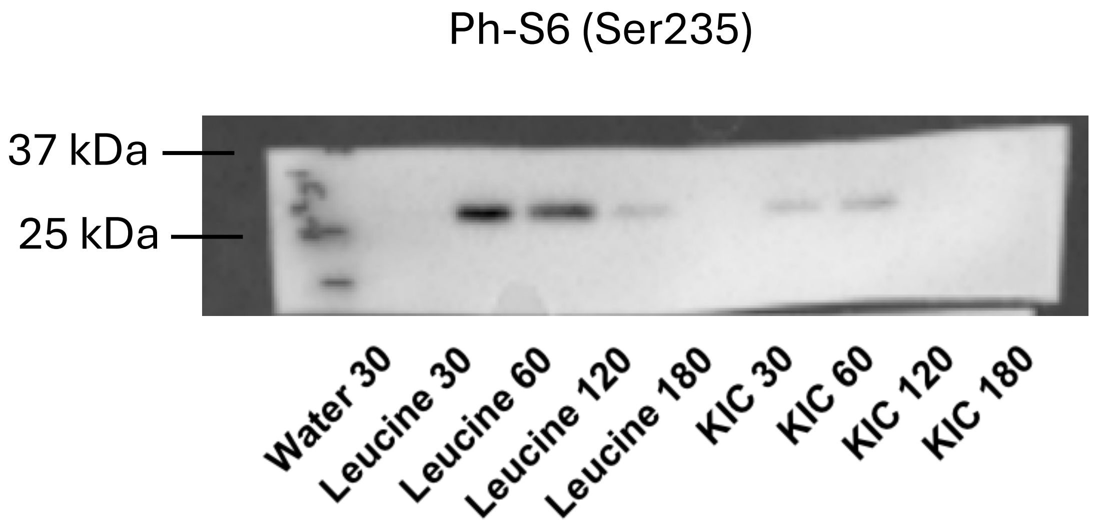

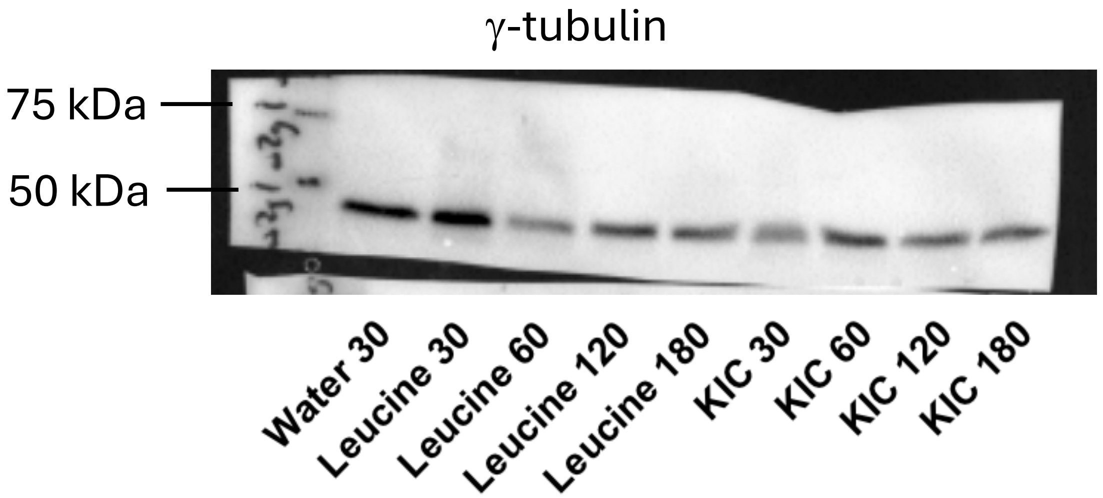

Blots for fig 3c

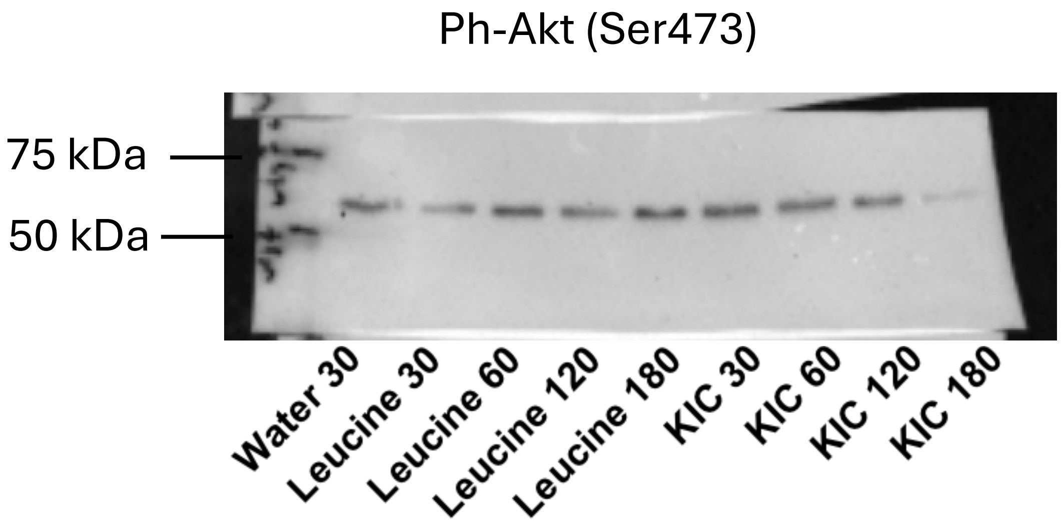

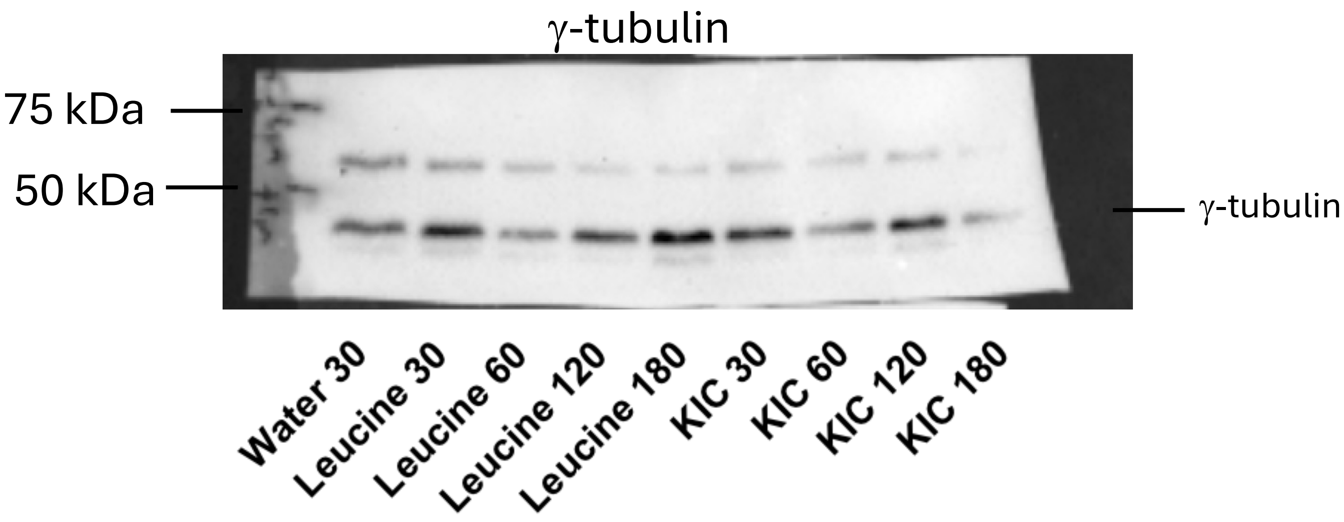

Blots for fig 3c

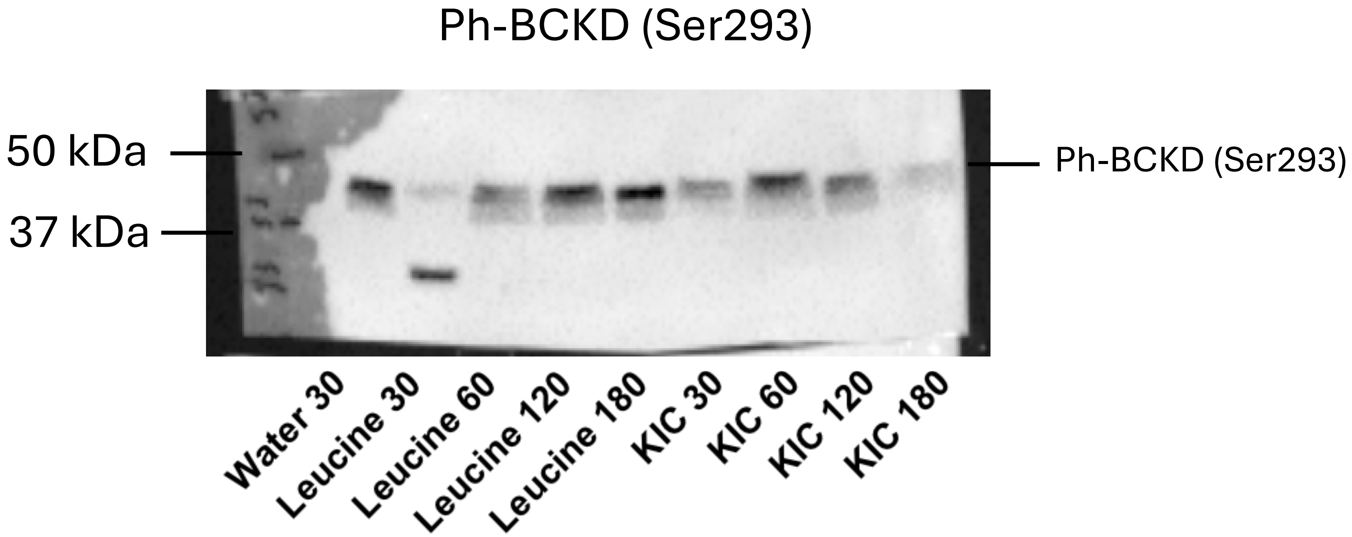

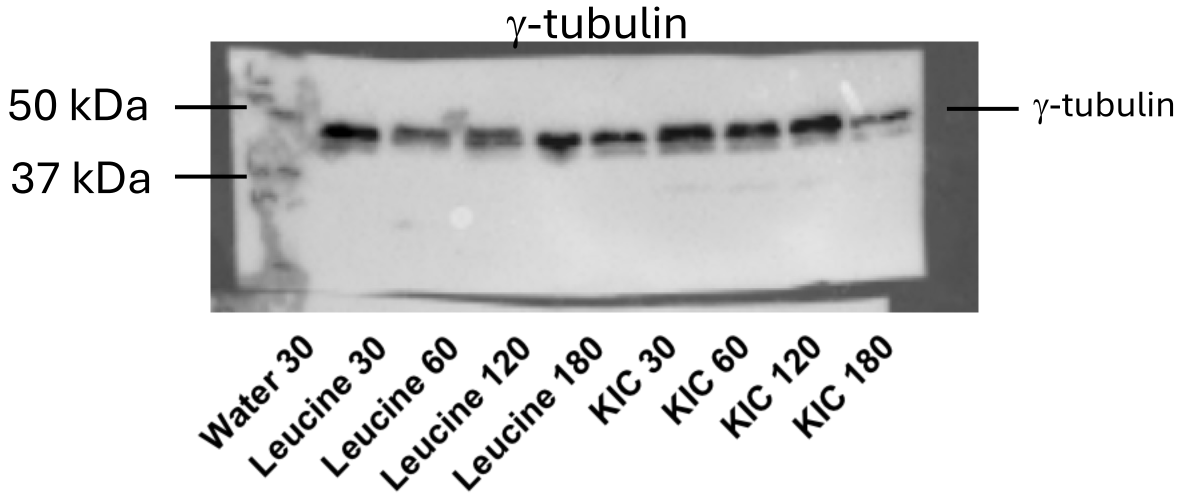

Blots for fig 4c

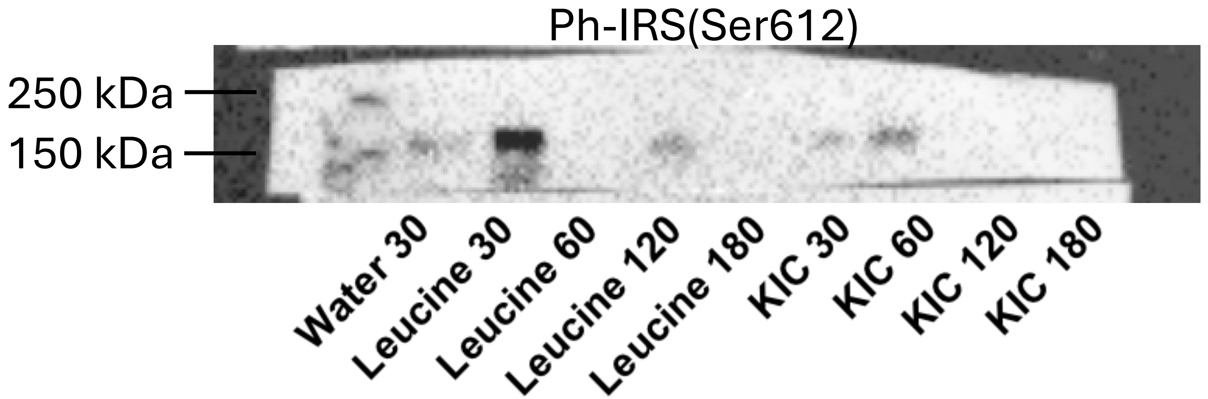

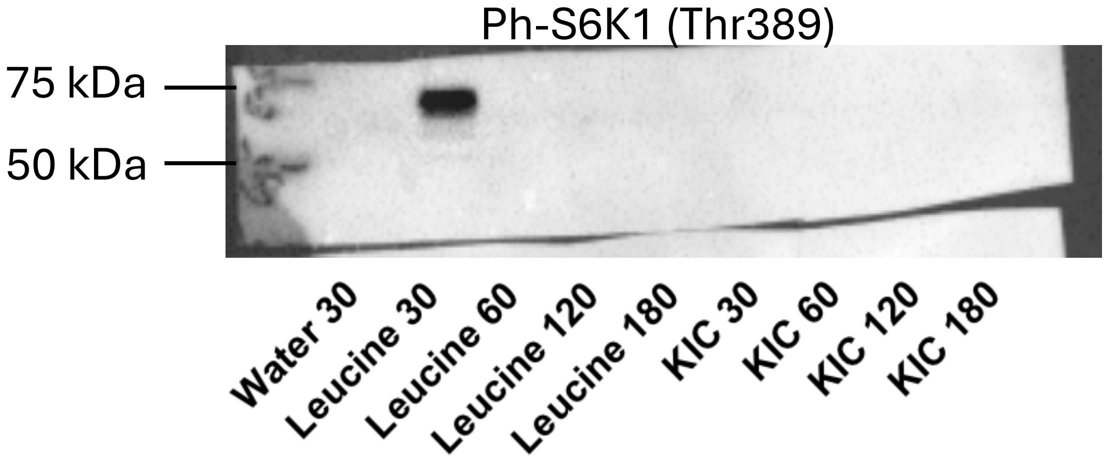

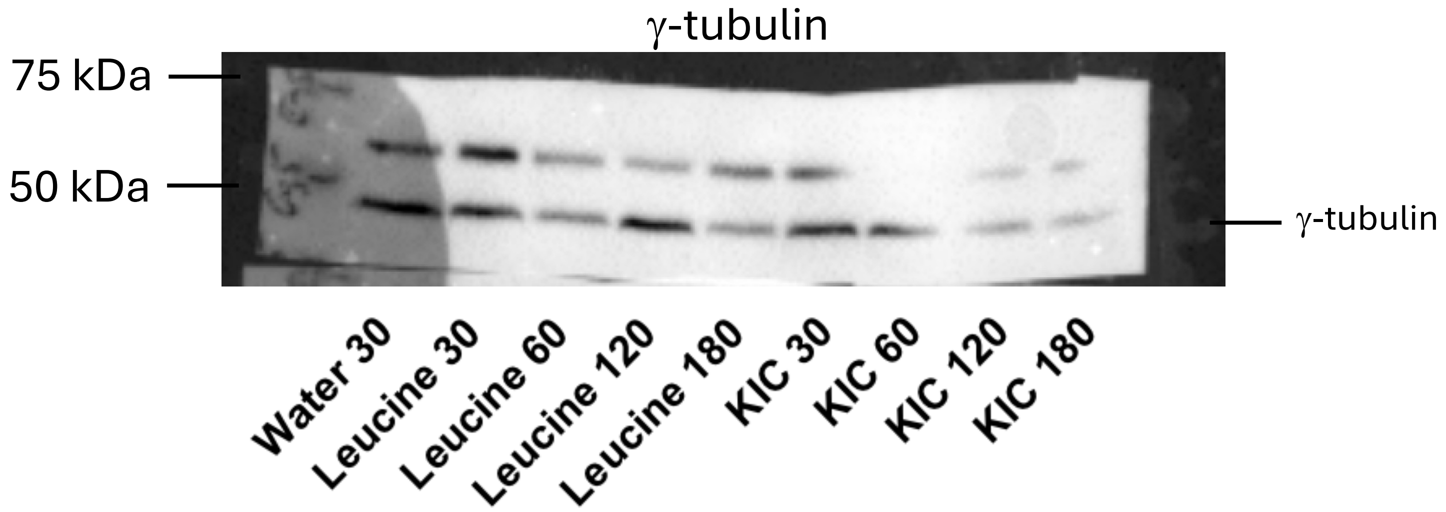

Blots for fig 4c

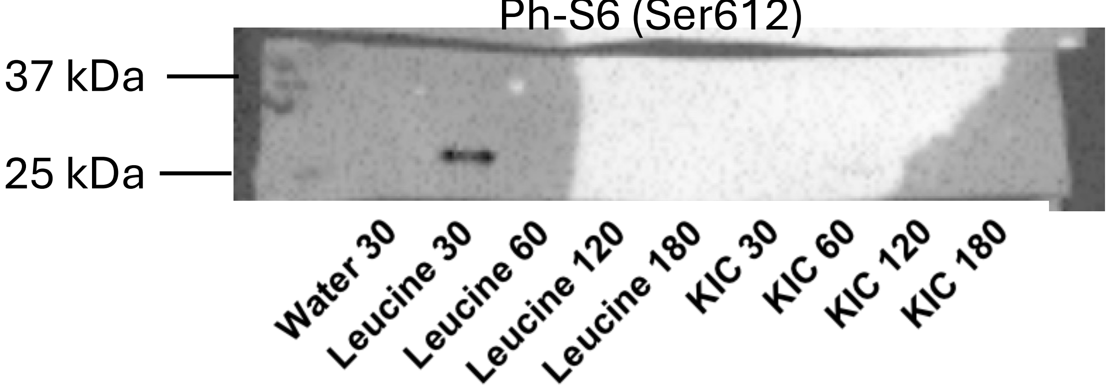

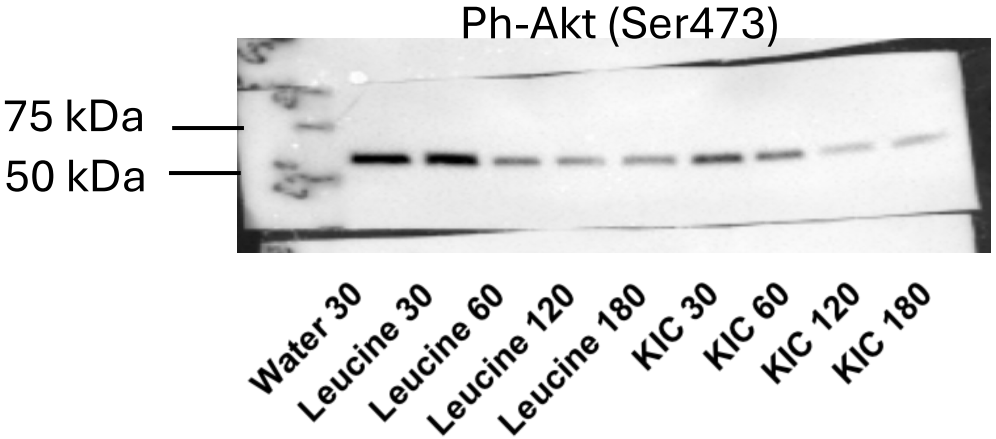

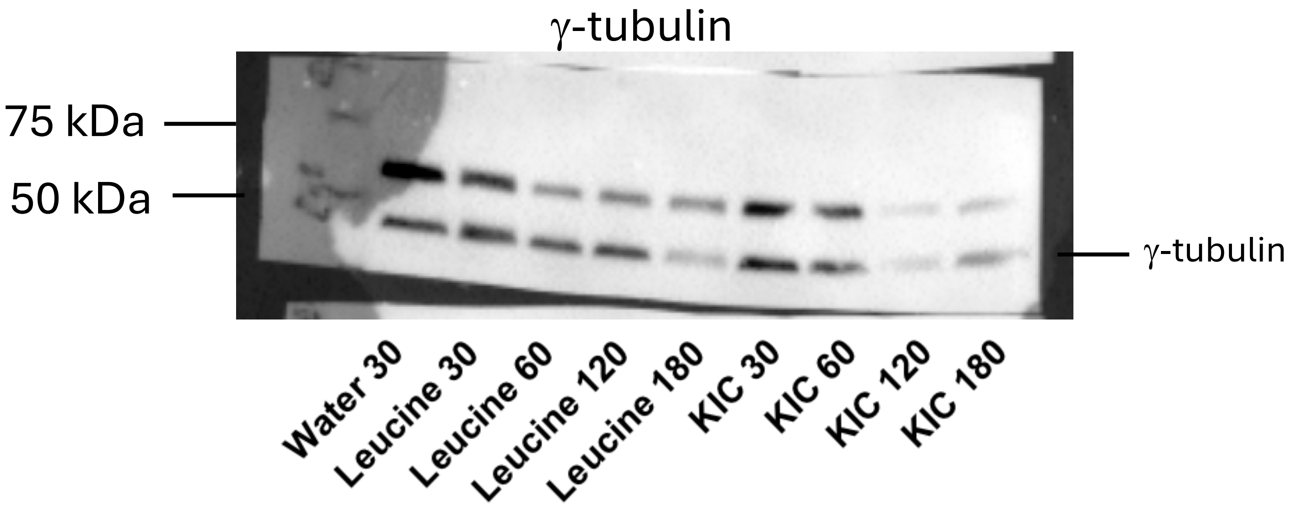

Blots for fig 4c

Ph-BCKD (Ser293)

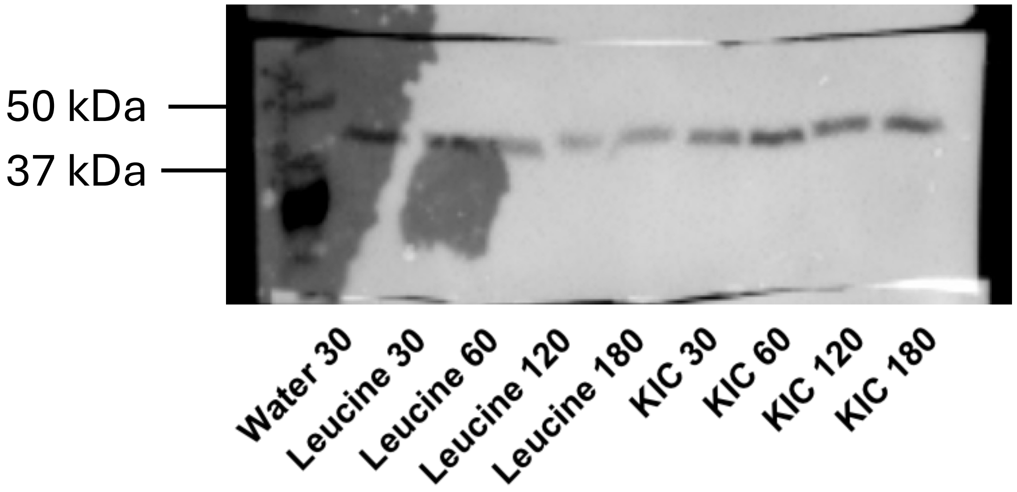

γ-tubulin

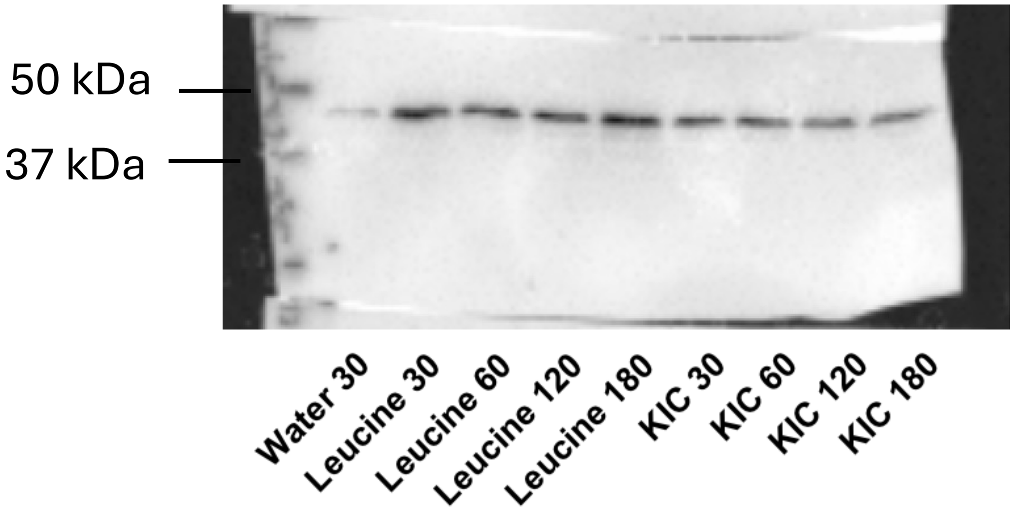

Blots for fig 5c

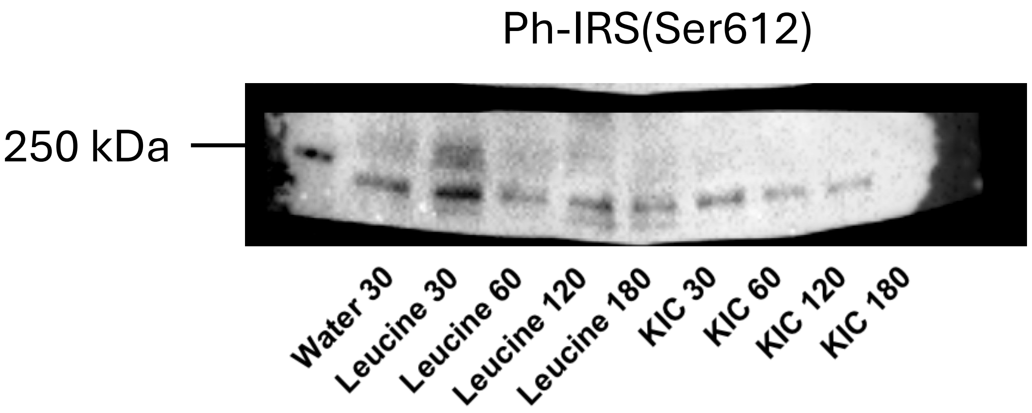

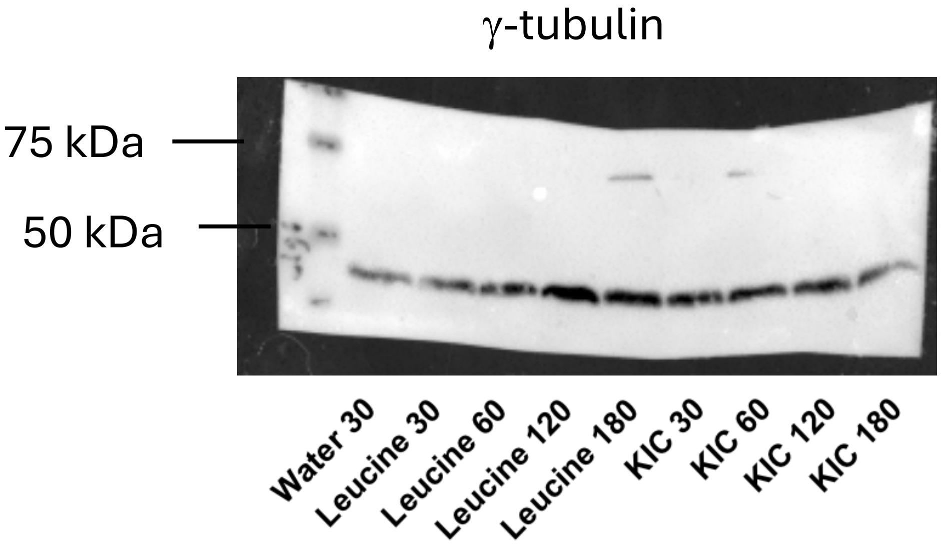

Blots for fig 5c

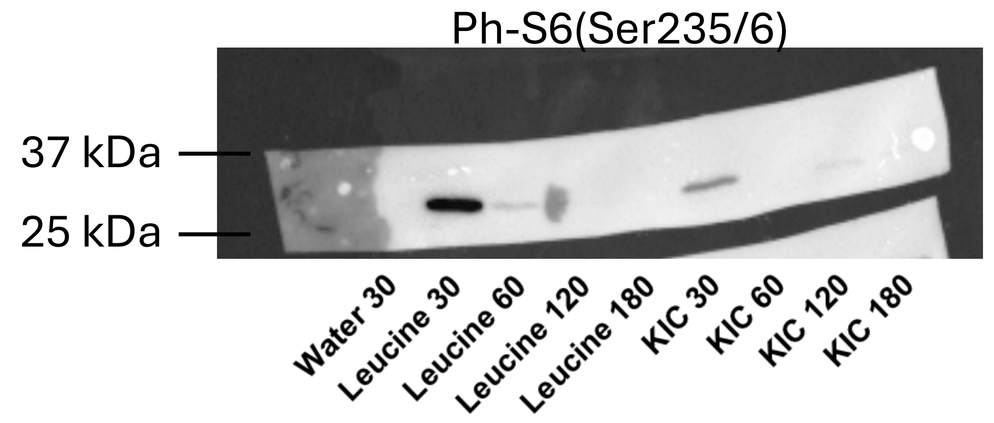

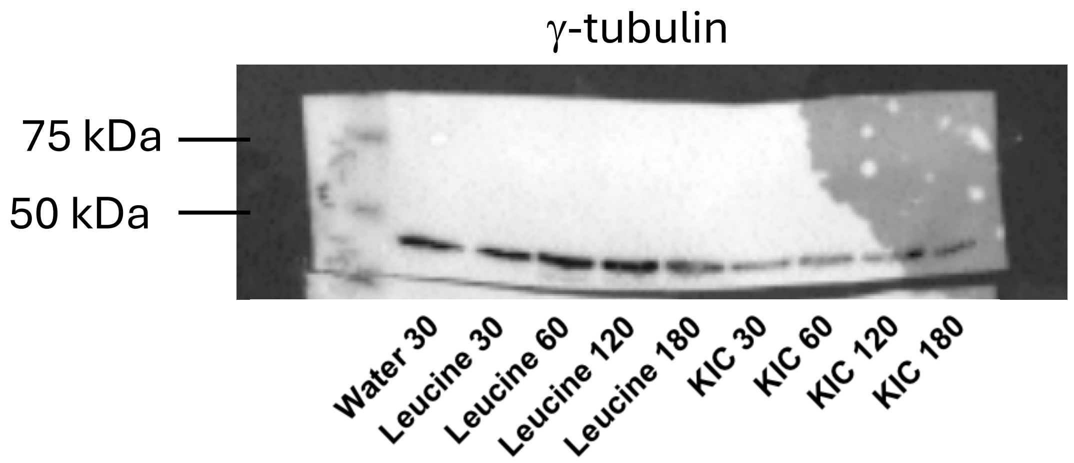

Blots for fig 5c

Ph-BCKD(Ser293)

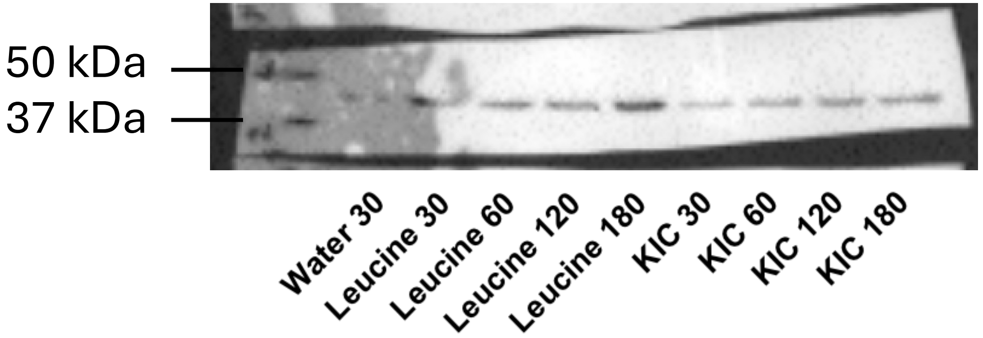

γ-tubulin

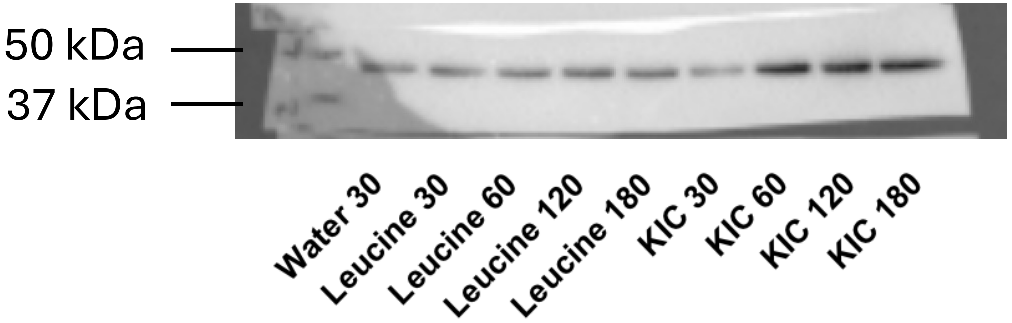

Blots for fig 6c

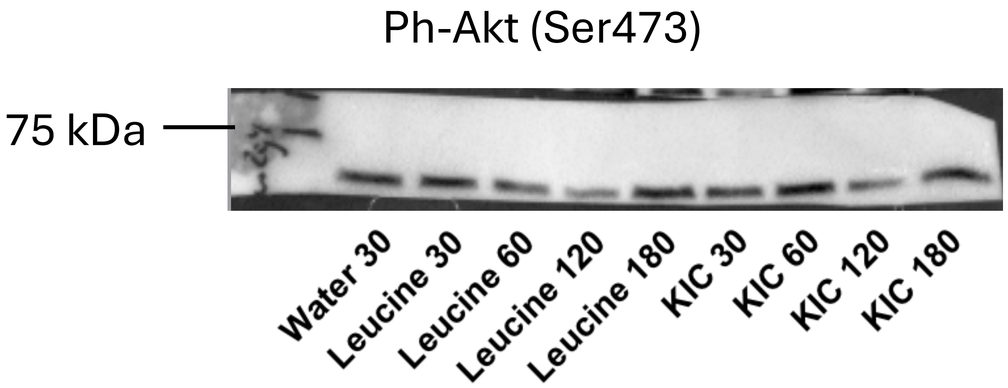

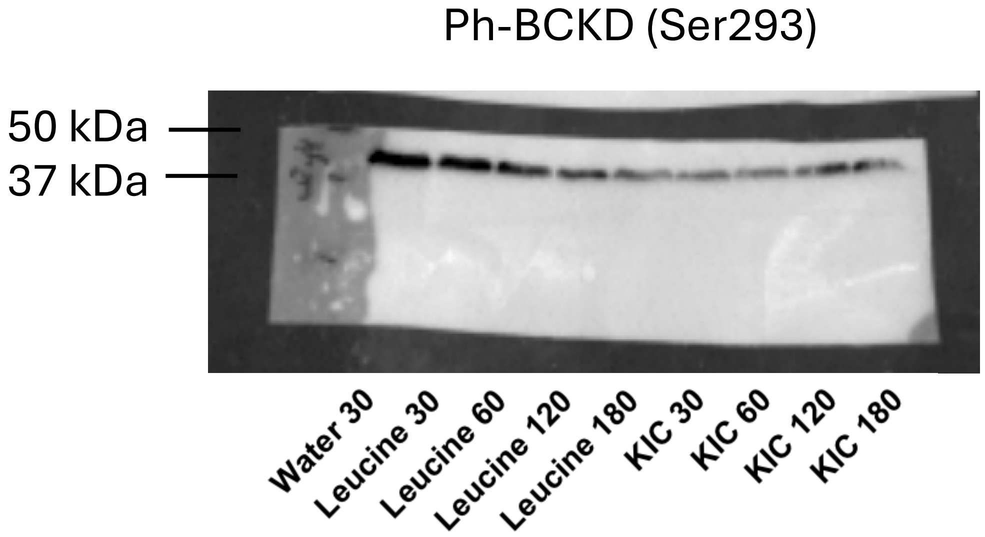

Blots for fig 6c

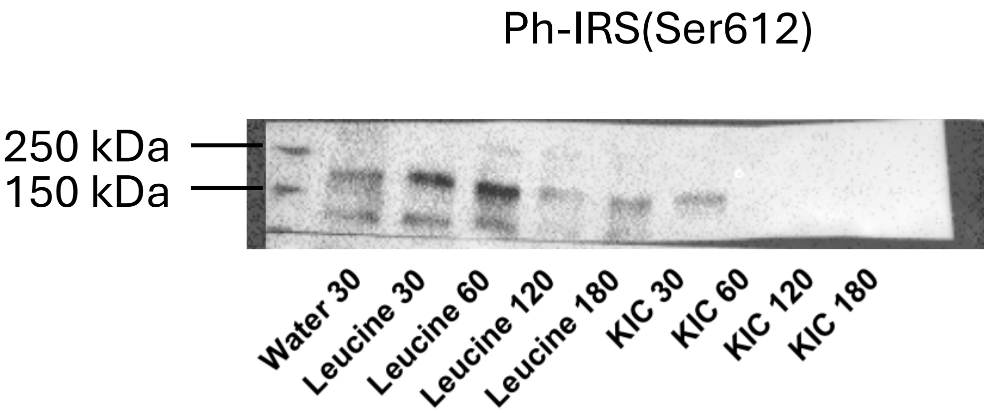

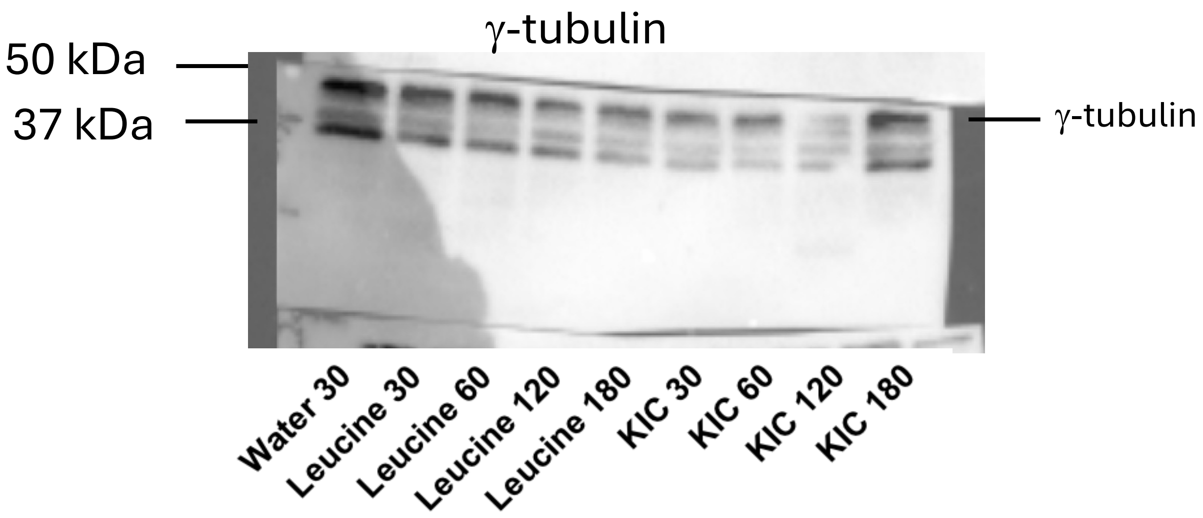

Blots for fig 6c

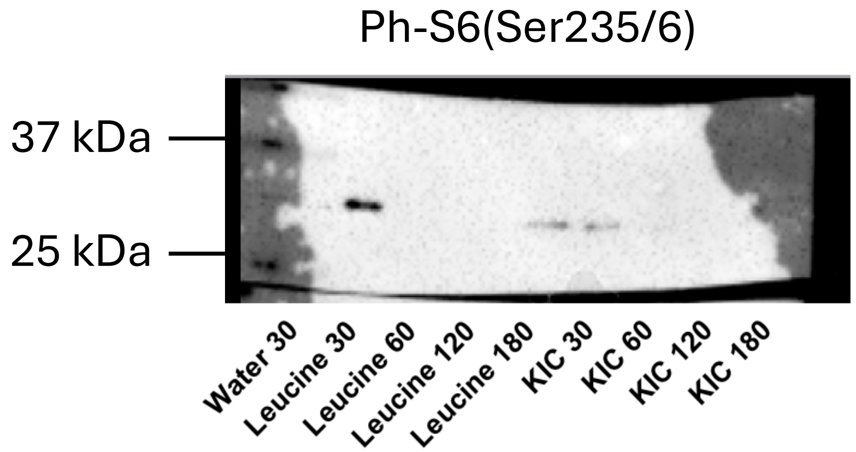

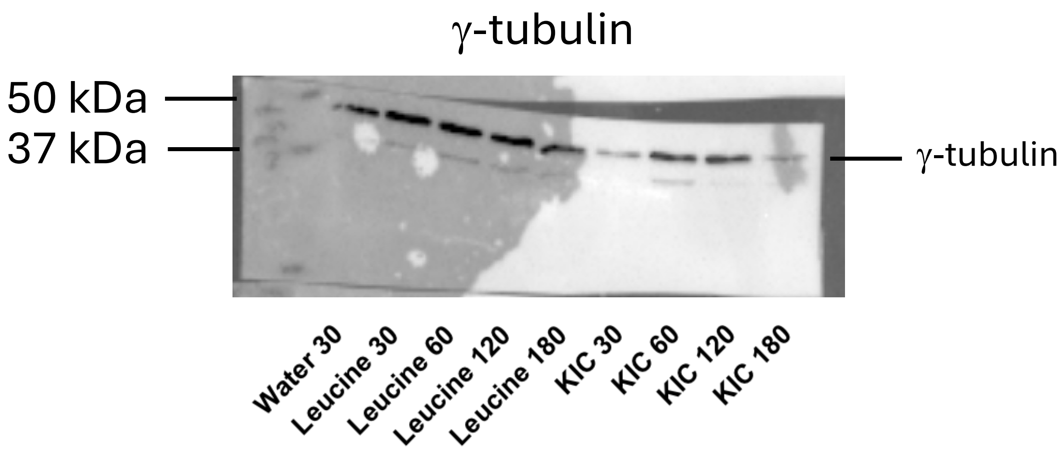

Blots for fig 7a

BCKD (first 5 lanes used for Fig 7A)

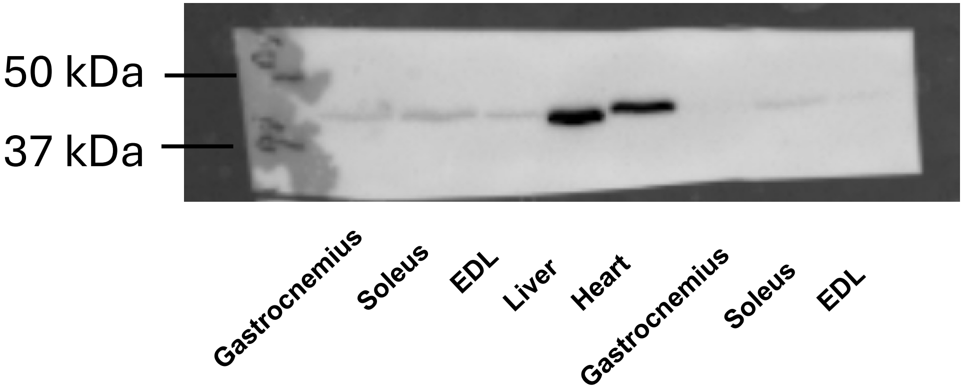

BDK (first 5 lanes used for Fig 7A)

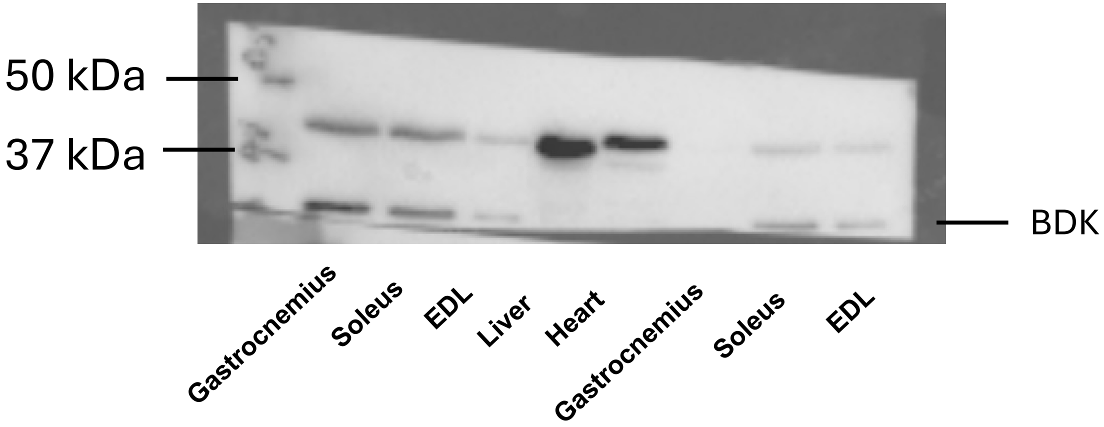

γ-tubulin (first 5 lanes used for Fig 7A)

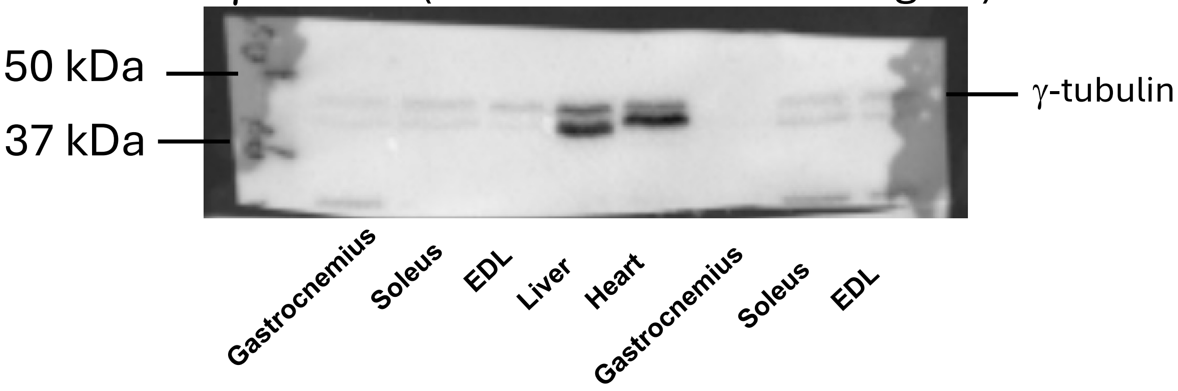

Blots for fig 7a

Ph-BCKD(Ser293) (last lane used for fig 7A)

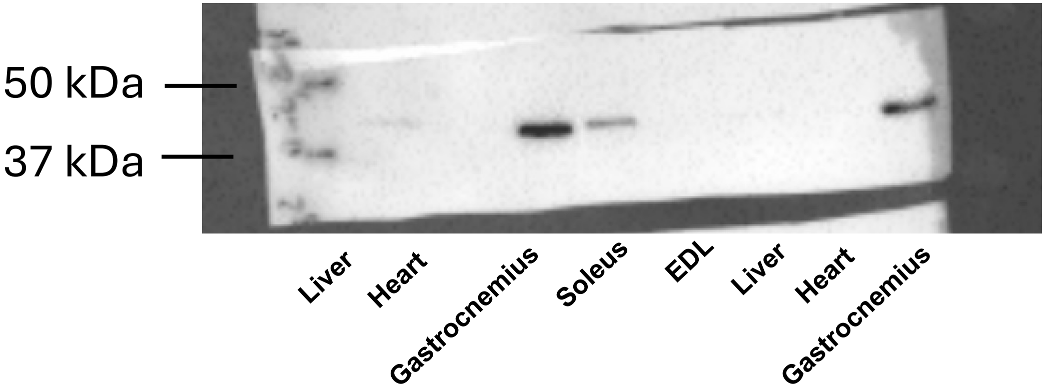

Ph-BCKD(Ser293) (first 4 lanes were used for fig 7A)

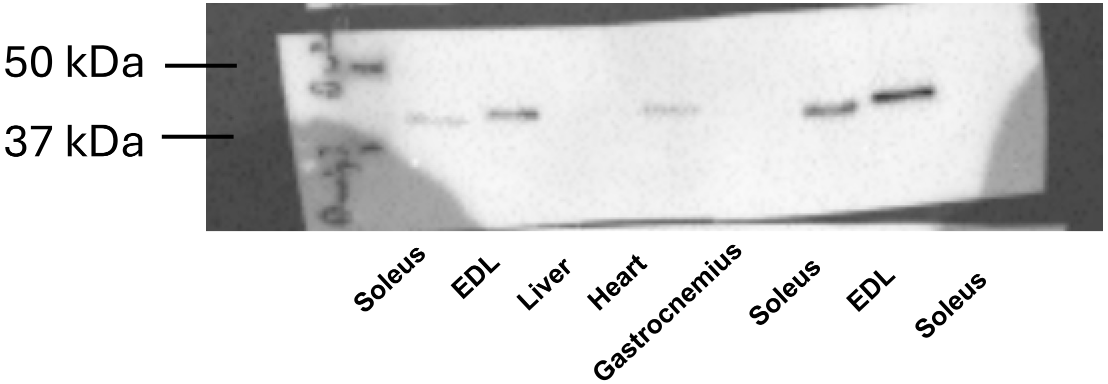

γ-tubulin (last lane used for fig 7A)

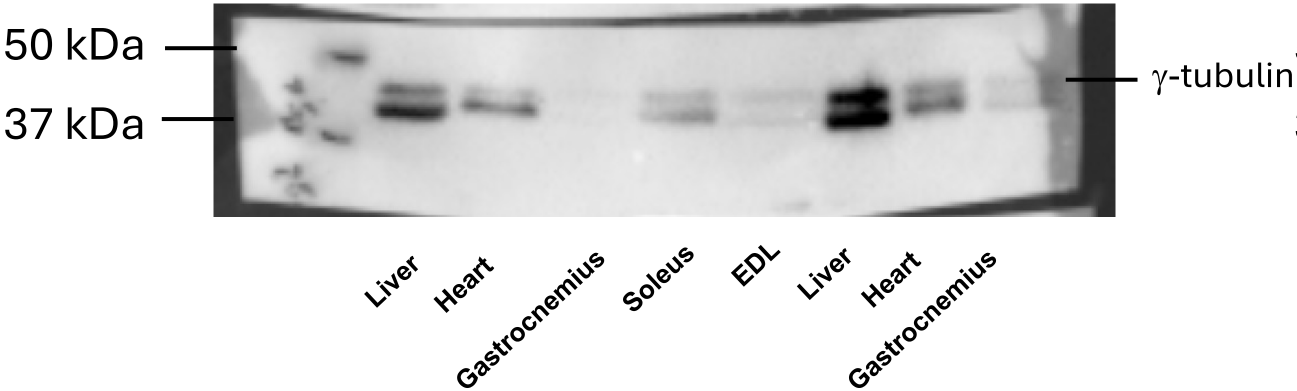

γ-tubulin (first 4 lanes were used for fig 7A)

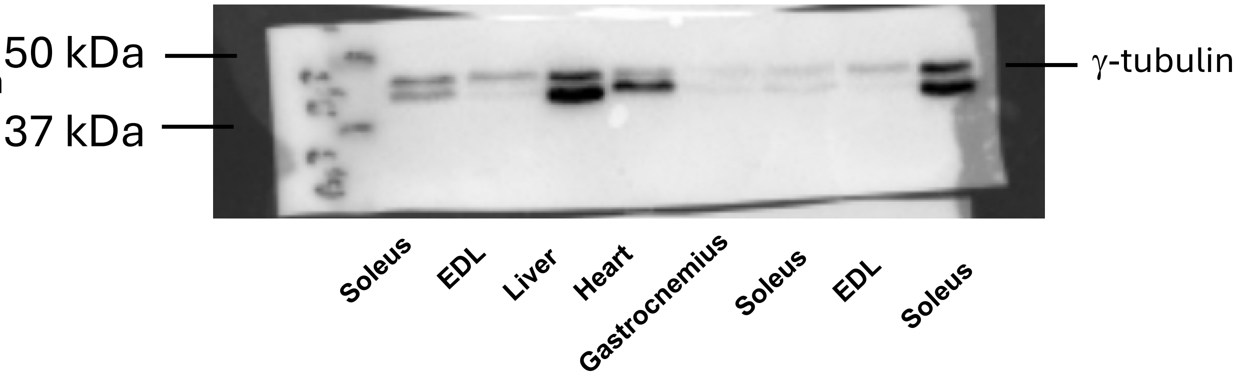

Blots for fig 7a

BCAT2 (first 3 lanes used for Fig 7A)

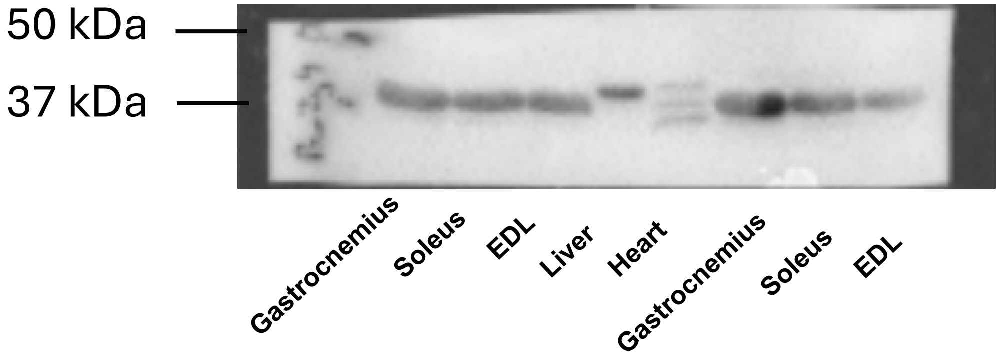

BCAT2 (first 2 lanes used for Fig 7A)

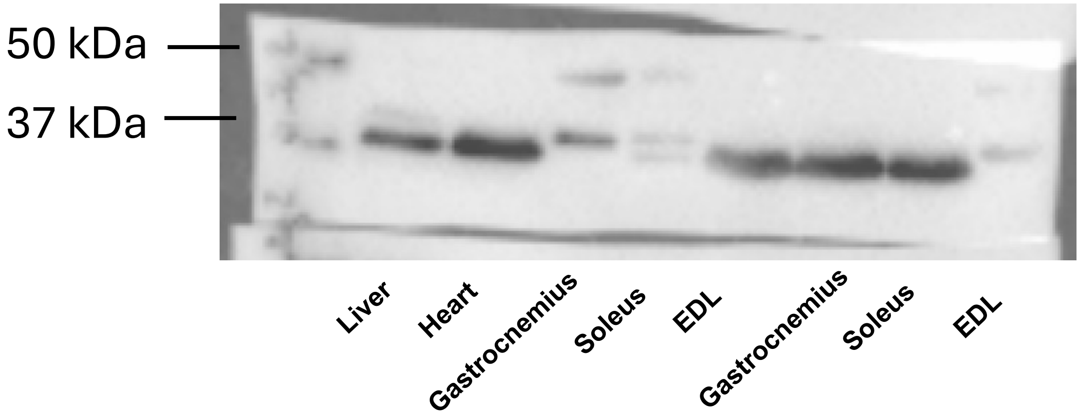

γ-tubulin (first 3 lanes used for Fig 7A)

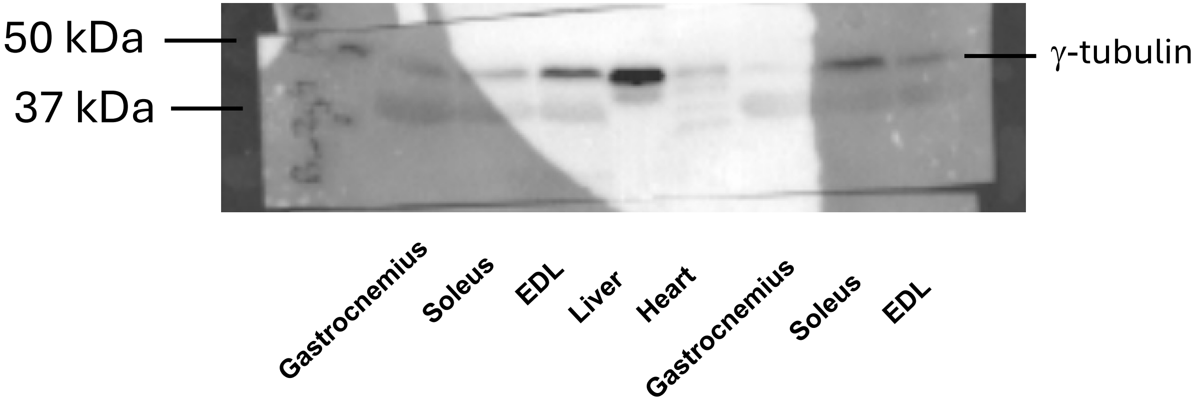

γ-tubulin (first 2 lanes used for Fig 7A)

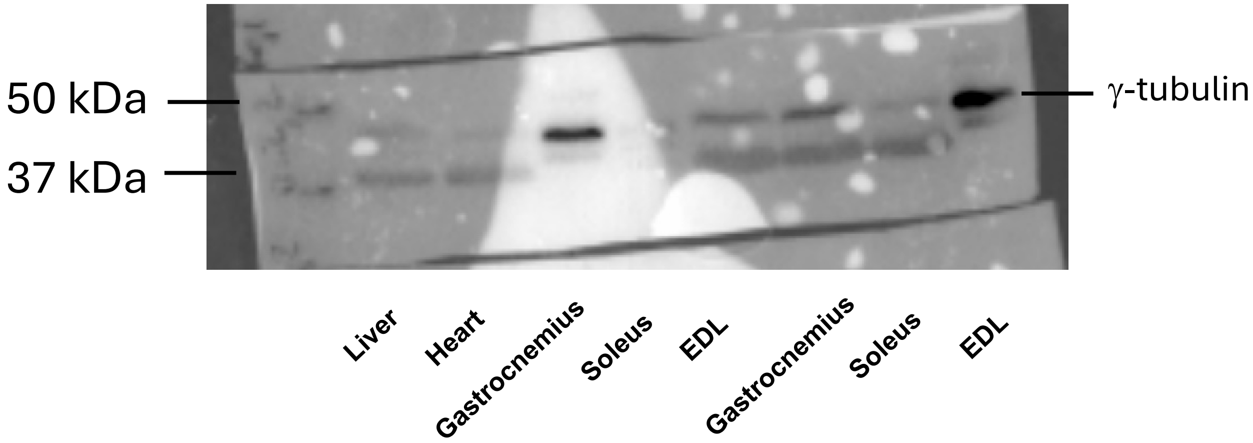

Blots for fig 7a

pp2Cm (first 5 lanes used for Fig 7A)

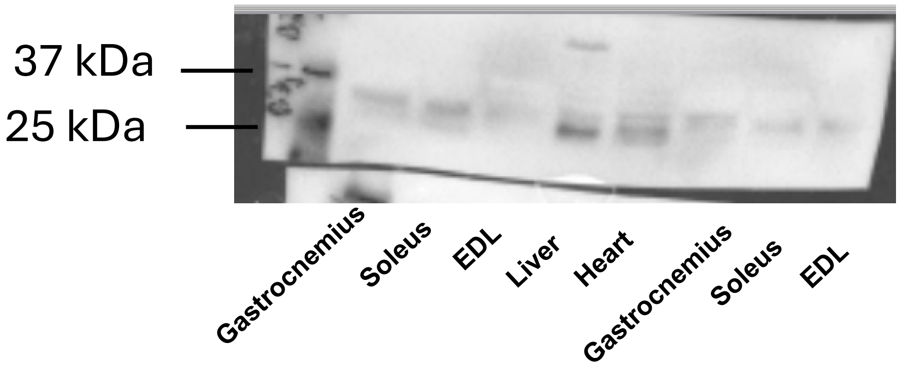

γ-tubulin (first 5 lanes used for Fig 7A)

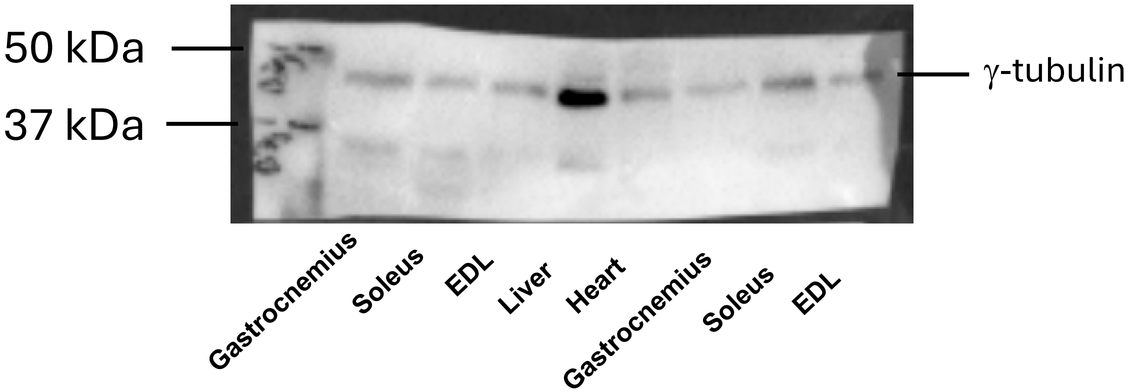

Additional Western blots in response to reviewers' comments

LIVER

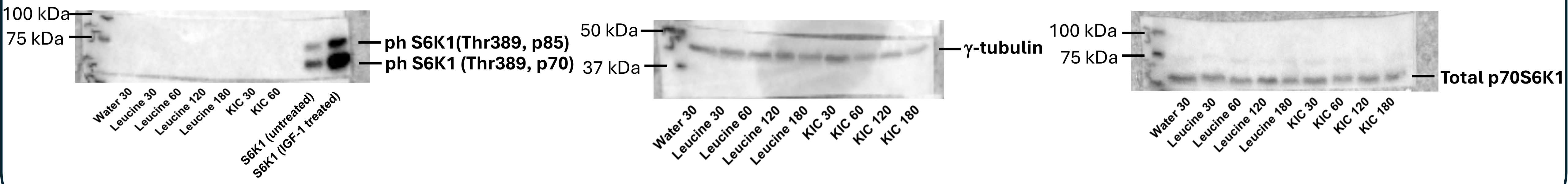

S6K1 (untreated and IGF-1 treated) are control S6K1 extracts

HEART

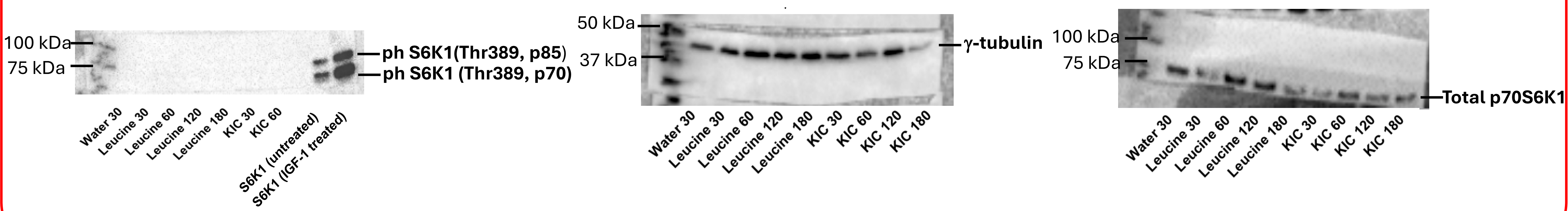

Supplement: S1 File — (PDF) [file pone.0309324.s004.pdf]
