# Supplementary material for: KIC (ketoisocaproic acid) and leucine have divergent effects on tissue insulin signaling but not on whole-body insulin sensitivity in rats
Source: PLoS One. 2024 Aug 20;19(8):e0309324. doi: 10.1371/journal.pone.0309324 (PMC11335129; doi:10.1371/journal.pone.0309324)
Supplement: Attachment — Submitted filename: Mann et al - response to reviewers-July19-24.docx [file pone.0309324.s006.docx]

We thank the associate editor and the reviewers for carefully reviewing the edits we made to our manuscript and the opportunity to fix this revision. We have thoroughly reviewed the manuscript to reflect the concern raised. In the response below, references to page and line numbers are for the clean copy of the revised manuscript.

Reviewer 2:

I thank the authors for their additional experiments and revisions from the previous review. For the most part all comments were addressed except for the following:
The authors clearly demonstrate that leucine and KIC levels are elevated at 30 minutes and significantly decrease in most tissues by 60 minutes following gavage, yet the insulin tolerance tests performed occurred at 60 minutes. The authors argue based on these tolerance tests that there is no insulin resistance caused by leucine and KIC, yet their time point is clearly after levels have returned to normal physiological levels and no longer match levels cited in their discussion as being pathological.

The response provided to this comment does not adequately address the concern provided. As demonstrated by the data provided, levels of circulating leucine concentrations decrease at 60min from the peak at 30min, and in tissues the levels return to the same as control (water gavage). The argument that leucine levels remaining at 100 umole/liter is still quite high and thus similar to those seen with obesity and insulin resistance contradicts the findings in the works cited. Citation 45 shows that circulating leucine levels in nonobese subjects were 112uM and citation 46 shows that in normoglycemic that it was 113uM. In both citations levels were above 120uM if not above 130uM in groups with obesity and/or insulin resistance, suggesting that there is likely a minimum threshold to impact physiology, a threshold not being met at 60 minutes. This implication is supported by your findings as levels dramatically reduce at the tissue level when circulating levels decrease below this threshold. It is completely reasonable based on the veterinary recommendation that this limitation can not be addressed via experimentation, but this needs to be addressed in the discussion as the current study only suggests that the effects of leucine are transient and likely concentration dependent, and not the conclusion reached in lines 491-495

**Authors’ Response**

We thank the reviewer for taking the time to read the revisions we made to the manuscript and for the reviewer’s comment. Although plasma leucine level was still significantly much higher at the time of the ITT (at least 3X the values in the water gavage group), we agree with the reviewer that there might be a threshold that is higher than what was observed at 60 min after gavage, especially in the tissues. Therefore, we have edited lines 491-498 (Discussion section) and lines 600-605 (Limitation section) to reflect this possibility. These sections now read:

Lines 491-498: “The fact that we observed no significant effect of leucine/KIC gavage on whole body insulin sensitivity in this study even with circulating levels of leucine and KIC that were much higher than basal (especially for leucine) suggests that altered leucine and KIC levels are a product of insulin resistance instead of causative. However, because the insulin tolerance test was carried out 60 min post gavage, at a time point when circulating and tissue levels of leucine and KIC were off their peak values (attained at 30 minutes), it is possible that the effects of leucine and KIC on insulin sensitivity are concentration dependent and transient.”

Lines 600-605: “Additionally, we performed the insulin tolerance test 60 min after gavage instead of at 30 min (when circulating and tissue leucine and KIC levels peaked). This decision was made based on the recommendation of the institutional animal care committee in order to reduce the stress of consecutive gavages/injections in a close time frame. By so doing however, we might have missed a critical time point because it is possible that the effect of the gavage on insulin sensitivity is concentration dependent and transient.”
